# To Be or Not to Be Eutectic: Anomalous Glass Forming Ability Governed by Melt Structural Complexity

**DOI:** 10.1002/advs.78359

**Published:** 2026-10-18

**Authors:** Wei Chen, Xuerui Wei, Chengzhe Wang, Zhichao Lu, Fan Zhang, Yan Huang, Yibo Zhang, Liang Wang, Saichao Cao, Chunyin Zhou, Ke Yang, Zhendong Fu, Jinkui Zhao, Dongbai Sun, Fanqiang Meng, Zhou Zhou, Xun‐li Wang, Weihua Wang, Dong Ma

**Affiliations:** ^1^ Center for Advanced and Ultrafast Photonic Science, School of Physics Sun Yat‐sen University Guangzhou China; ^2^ Center for Neutron Science and Technology, Guangdong Provincial Key Laboratory of Magnetoelectric Physics and Devices, School of Physics Sun Yat‐sen University Guangzhou China; ^3^ Dongguan Institute of Materials Science and Technology Chinese Academy of Sciences Dongguan China; ^4^ Neutron Science Center Songshan Lake Materials Laboratory Dongguan China; ^5^ Sino‐French Institute of Nuclear Engineering and Technology Sun Yat‐Sen University Zhuhai China; ^6^ Department of Physics City University of Hong Kong Hong Kong China; ^7^ Shanghai Synchrotron Radiation Facility, Shanghai Advanced Research Institute Chinese Academy of Sciences Shanghai China; ^8^ Southern Marine Science and Engineering Guangdong Laboratory Zhuhai China; ^9^ School of Materials Science and Engineering Sun Yat‐sen University Guangzhou China

**Keywords:** aerodynamic levitation, alloy melt, atomic structure, metallic glass, synchrotron x‐ray diffraction

## Abstract

For decades, the search for metallic glass formers has followed a simple rule: go for the eutectics. However, this conventional wisdom has been challenged by a lingering mystery—certain congruent melting alloys exhibit higher glass‐forming ability (GFA) than their eutectic neighbors. Here, we unravel this anomaly by probing the structure of high‐temperature melt—the direct precursor to a glass. Using Ti‐Cu as a model system, we conduct a comparative study on a eutectic Ti_27_Cu_73_ and its congruent melting counterpart Ti_50_Cu_50_. By combining aerodynamic levitation, in situ synchrotron x‑ray diffraction, and an energy minimization‐enhanced reverse Monte Carlo method, we resolve the atomic structures of these alloys in both the melt and glassy states. We reveal that the congruent melt is structurally more complex than the eutectic, exhibiting higher cluster diversity and stronger inter‑cluster correlations owing to the entropy‐enthalpy competition. This structural complexity is then inherited and amplified during the glass transition via cluster transformations, serving as dual kinetic and energetic barriers to crystallization that lead to the anomalously higher GFA. Our work establishes melt structural complexity as a new framework for understanding the glass transition at the atomic cluster level, with important implications for designing superior glasses from high‐entropy intermetallic alloys.

## Graphical abstract

Challenging the eutectic rule, congruently melting Ti–Cu alloys show greater glass‐forming ability than their eutectic counterparts. This arises from the congruent melt's structural complexity: higher cluster diversity and stronger inter‐cluster correlations from entropy–enthalpy competition. This complexity is inherited and amplified across the glass transition, creating dual kinetic and energetic barriers to crystallization, yielding anomalous GFA.

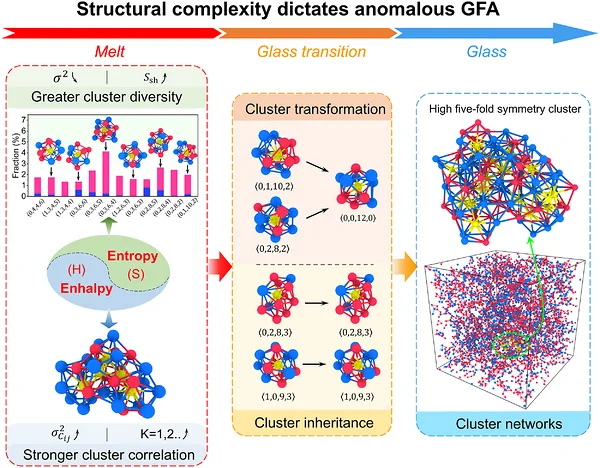

## 1 Introduction

Metallic glasses (MGs), also known as amorphous alloys, are formed by rapidly cooling high‑temperature melts (HTMs) to bypass crystallization. Their disordered atomic structure imparts exceptional properties, including high strength, high elastic limit, and soft magnetism. These enable important applications in consumer electronics, energy conversion, and aerospace industries, such as amorphous hinges for foldable screen phones and amorphous magnetic cores for transformers [1, 2, 3, 4, 5, 6, 7]. For these applications, glass‑forming ability (GFA) is a critical factor that governs component size, quality, and cost. Therefore, understanding glass transition and GFA is essential for the development of superior metallic glasses.

For more than half a century, a simple golden rule has guided the search for better metallic glasses: follow the eutectics [8]. This can be traced back to 1960, when Duwez and colleagues [9] first fabricated a MG from the eutectic Au‐25%Si using rapid quenching. Soon after, eutectic compositions in systems such as Au‐Sn, Pd‐Si, and Pt‐Si were found to readily form MGs [10, 11]. In 1969, Turnbull provided a theoretical foundation for these observations, proposing the *T_rg_* criterion based on classical nucleation theory, where  *T_rg_* = *T_g_* /*T_l_*, with *T_g_* the glass transition temperature and *T_l_* the liquidus temperature [12]. A higher *T_rg_* implies a lower nucleation rate and thus better GFA. Since eutectic alloys possess the lowest melting temperatures and *T_g_* varies little with composition, they naturally maximize *T_rg_* and thus exhibit the best GFA. Guided by this criterion, targeting eutectic compositions became the prevailing paradigm for discovering new MGs [13, 14, 15]. However, in 2005 and later, the discovery of several outlier metallic glasses in binary systems such as Zr‐Cu [16], Hf‐Cu [17], Ti‐Cu [18], and Ni‐Nb [19] began to defy this convention. In these systems, the compositions with the highest GFA were not the low‐melting eutectics, but rather congruent melting compositions (e.g., Zr_50_Cu_50_) or off‐eutectic compositions (e.g., Zr_36.5_Cu_63.5_). The *T_rg_* criterion, along with other kinetic models [12], could not explain these experimental observations, giving rise to what we term anomalous GFA—a departure from the classical eutectic rule. To be or not to be *eutectic*: that is now the question.

To address this dilemma, Wang et al. [20] invoked the concept of metastable deep eutectics proposed by Greer et al. [21]. They argued that complex intermetallic compounds in an equilibrium phase diagram could be kinetically suppressed during rapid cooling, thus creating a nonequilibrium phase diagram with a metastable deep eutectic. The equi‐atomic composition or the congruent melting composition sits near this metastable eutectic point, thereby yielding a higher equivalent *T_rg_* and enhanced GFA [20]. This rationale appears to work well for systems like Zr‐Cu, but it is not universal—Zr‐Ni, for instance, exhibits a much deeper metastable eutectic than Zr‐Cu yet displays far poorer GFA [22]. Meanwhile, a eutectic coupled zone model has been employed to explain optimal glass formation at off‐eutectic compositions [16, 23, 24], but it fails to address why the highest GFA also emerges at congruent melting compositions. Thus, despite these efforts, the origin of this anomalous GFA remains elusive—a mystery that lingers up to date.

The foregoing efforts have all focused on crystallization kinetics—how to suppress competing crystalline phases. However, they have largely overlooked the possibility that the melt itself may hold the key to its own resistance against crystallization. After all, the high‐temperature melt is the common precursor to both the glass and its crystalline competitors; its structure and stability must therefore be intimately linked to glass‐forming ability [25, 26, 27, 28]. This perspective builds on a rich history of insights. For instance, in a seminal study in 1965, Adam and Gibbs introduced the concept of cooperatively rearranging regions, revealing how configurational entropy governs relaxation dynamics and viscosity in glass‐forming liquids [29, 30]. Angell later captured this temperature‐dependent viscosity behavior in the classic Angell plot, showing that melt fragility serves as a measure of liquid stability—a property that correlates intimately with GFA [31]. From a structural perspective, diffraction experiments and simulations have shown that medium‐range order (MRO) in the glass‐forming melt, beyond the first coordination shell, correlates closely with glass‐forming tendency. Using high‐temperature XRD, Hu et al. showed that in Au–Cu–Si, the inheritance of MRO from the melt to the glass is key to enhanced GFA [32]. Lan et al. combined time‐resolved synchrotron measurements of an electrostatically levitated glass‐forming liquid (Zr_57_Nb_5_Al_10_Cu_15.4_Ni_12.6_) with molecular dynamics simulations, revealing that short‐to‐medium‐range order grows upon cooling as a result of increased correlation between solute atoms [33]. M.Z. Li et al. [28, 34] used molecular dynamics simulations to reveal a string‐like backbone of icosahedral clusters and hidden MRO features inherited from crystalline order, whose topological entanglement correlates with GFA. More recently, simulations by Wang, Flores, and colleagues demonstrated that structural signatures of GFA, such as cluster diversity and local ordering, are indeed encoded within the alloy melt itself [35, 36]. This is also supported by Cao et al., who demonstrated that the addition of La to Al–Cu melts does not promote icosahedral short‑range order but increases cluster diversity and reduces cluster fraction variance, leading to a more disordered melt structure and, consequently, greater GFA [37]. Clearly, quantifying melt structure in terms of clusters and their connectivity—that is, medium‐range order—is essential for understanding the GFA anormaly.

Inspired by these earlier investigations, we turn to the high‐temperature melt—the precursor to the glass—to address the GFA dilemma from the perspective of melt structure and stability. First, using Ti‐Cu as a model system, we demonstrate that the same GFA anomaly exists: the high‐melting congruent compound Ti_50_Cu_50_ exhibits greater GFA than the low‐melting eutectic composition Ti_27_Cu_73_. Second, to uncover the structural origin of this anomaly, we combine laser‐heated aerodynamic levitation with in situ high‐energy synchrotron x‐ray diffraction (Figure S1a) and a newly developed energy minimization‐enhanced reverse Monte Carlo (EM‐RMC) method (Figure S1b), providing direct access to the atomic‐scale structures of the high‐temperature melts and their corresponding glasses. Third, we employ Voronoi analysis to quantify atomic cluster types, fractions, and spatial distributions, and introduce two new parameters—cluster Shannon entropy and cluster correlation variance—to capture cluster diversity and cluster connectivity, respectively.

Our results reveal that the congruent melt is essentially structurally more complex than its eutectic counterpart, marked by both greater cluster diversity and stronger inter‐cluster correlations. This structural complexity, driven by entropy–enthalpy competition, is inherited and further enhanced via cluster transformation during the glass transition, acting as both kinetic and energetic barriers that suppress crystal nucleation. Together, these findings uncover the physical origin of the anomalous GFA and offer a fresh perspective for understanding the glass transition and GFA at the atomic cluster level.

## 2 Results

The Ti–Cu system offers a particularly clean realization of the GFA anomaly. As will be described below, the GFA of Ti_50_Cu_50_ exceeds that of the neighboring eutectic composition (Ti_27_Cu_73_), with Ti_50_Cu_50_ lying at an apparently hypothetical metastable deep eutectic point [20, 21]. This metastable eutectic is more ideal than those in the Zr–Cu and Zr–Ni systems [20], as it is formed by extending the liquidus lines of the two terminal solid solution phases (β‐Ti and Cu) across the entire phase diagram, so that the only crystalline phases competing with glass formation are two simple terminal solid solutions. This isolates the role of melt structure from the confounding influence of multiple intermetallic competitors, making Ti–Cu an ideal model system to uncover the atomistic origin of anomalous glass formation from the melt structure.

### 2.1 Anomalous GFA and Characterization of Melt Structure

As shown in the phase diagram of Figure 1a, we selected two representative alloys in the Ti‐Cu binary system—the eutectic alloy Ti_27_Cu_73_ (denoted EA) and the congruent melting alloy Ti_50_Cu_50_ (denoted CMA)—for a comparative study. According to classical nucleation theory, EA is expected to possess greater GFA than CMA, but our experimental findings contradict this prediction. First, XRD patterns of melt‐spun ribbons prepared at a wheel speed (*v*) of 35 m/s (Figure 1b) show that CMA exhibits a diffuse scattering pattern, indicative of a fully amorphous structure. In contrast, the EA ribbons made at *v* = 40 m/s display a few weak crystallization peaks, and fully amorphous EA ribbons can be only obtained until increasing *v* up to 43 m/s. Second, differential scanning calorimetry (DSC) was used to obtain the heating curves of both alloys and determine the characteristic temperatures, including the glass transition temperature (*T_g_*), the crystallization temperature (*T_x_*) and the liquidus temperature (*T_l_*), as summarized in Table 1. Also listed in Table 1 are the values of conventional GFA indicators such as *T_rg_*, Δ*T_x_* (=*T_x_*  − *T_g_*), and γ (= *T_x_* /(*T_g_* + *T_l_*)) [38]. Notably, the DSC curve of EA shows no distinct *T_g_* (Figure 1c), further indicating its poorer GFA.

**FIGURE 1:**
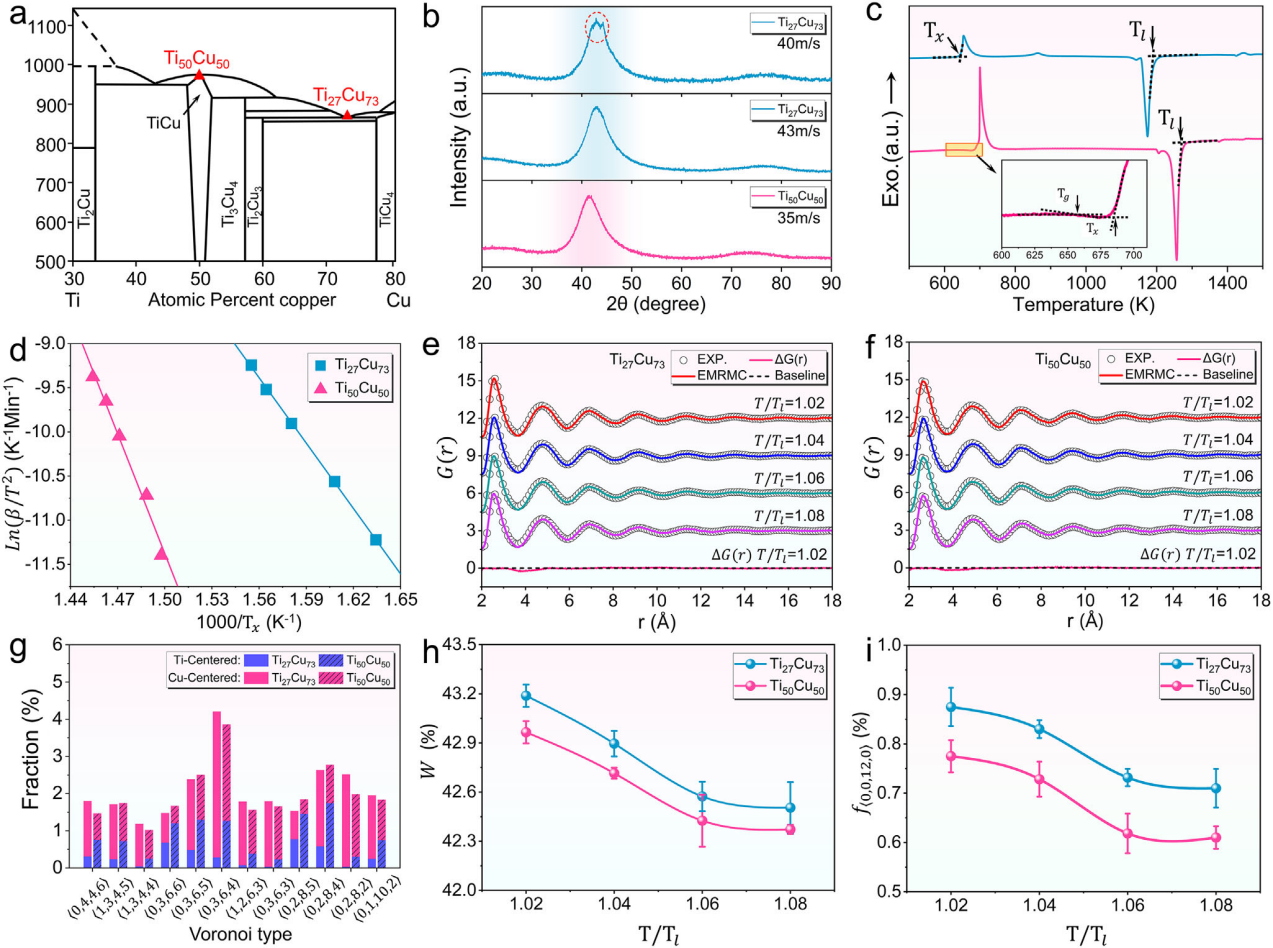
Experimental evidence for the anomalous GFA and melt structure characterization of Ti‐Cu alloys. (a) Ti‐Cu binary phase diagram [40] showing the eutectic alloy Ti_27_Cu_73_ (EA) and congruent melting alloy Ti_50_Cu_50_ (CMA). (b) XRD patterns of melt‐spun ribbons, indicating that CMA is fully amorphous at a wheel speed of 35 m/s, whereas EA requires a higher speed of 43 m/s to achieve a fully amorphous state. (c) DSC curves obtained at a heating rate of 30 K/min for Ti‐Cu MGs, revealing a distinct *T_g_* for CMA but no detectable *T_g_* for EA. (d) Kissinger analysis, showing a substantially higher crystallization activation energy for CMA than for EA. Together, these results demonstrate that the high‐melting CMA possesses greater GFA compared to the low‐melting eutectic EA. (e, f) Pair distribution functions *G*(*r*) of aerodynamically levitated Ti_27_Cu_73_ and Ti_50_Cu_50_ melts at different reduced temperatures (*T*/*T_l_*), respectively, where T*_l_* is the liquidus temperature. Open circles are the experimental data, solid lines are the EM‐RMC results, magenta lines denote Δ*G*(*r*), and dashed lines indicate the baselines. Shown in (g) is a comparison of their Voronoi cluster distributions, distinguishing Ti‐ and Cu‐centered clusters. (h) and (i) show the temperature dependence of the average degree of fivefold local symmetry (*W*) and the fraction of 〈0, 0, 12, 0〉 clusters (*f*_〈0,0, 12, 0〉_), respectively, for the Ti_27_Cu_73_ and Ti_50_Cu_50_ melts.

**TABLE 1:** Characteristic parameters of Ti‐Cu MGs.

MG	*T_g_* (K)	*T_x_* (K)	*T_l_* (K)	Δ*T_x_* (K)	*T_rg_*	γ	Δ*E_x_* (kJ/mol)	*v_c_* (m/s)
Ti_27_Cu_73_	—	639.2	1190.9	—	—	—	205.3	≥43
Ti_50_Cu_50_	654.8	683.7	1274.3	28.9	0.514	0.354	376.1	≤35

Finally, the Kissinger method was used to evaluate the crystallization activation energies of the two alloys (Figure 1d) based on the following equation [39]:

(1) $$\ln\left(\frac{\beta }{T_{x}^{2}}\right)=\frac{\Delta E_{x}}{RT_{x}}+constant$$

where β is the heating rate (K/min), Δ*E_x_* is the crystallization activation energy (kJ/mol), and *R* is the gas constant. The *T_x_* values acquired at different heating rates are shown in Figure S2 and Table S1. The experimentally determined Δ*E_x_* of CMA (376.1 kJ/mol) is significantly higher than that of EA (205.3 kJ/mol), suggesting stronger resistance to crystallization. Consequently, our results demonstrate that the low‐melting eutectic alloy exhibits poorer GFA than the high‐melting congruent alloy.

To understand the origin of the anomalous GFA from the perspective of melt structure, we resolved three‐dimensional atomic structures of the two alloy melts at elevated temperatures by combining laser‐heated aerodynamic levitation, in situ high‐energy synchrotron X‑ray diffraction, and our newly developed energy minimization‐enhanced reverse Monte Carlo (EM‐RMC) method (*see* Methods). Figure 1e,f shows the pair distribution functions *G*(*r*) determined by in situ synchrotron x‐ray diffraction of aerodynamically levitated melts of EA and CMA, respectively, at various reduced temperatures (*T*/*T_l_*). The corresponding structure factors *S*(*q*) are displayed in Figure S3. Although the second scattering peak and its shoulder appear to evolve similarly for the two melts when plotted comparatively, differential analysis reveals distinct behaviors. Using *S*(*q*) at *T*/*T_l_* = 1.02 as a baseline, we calculated $\Delta S\;\left(q\right)=S{\left(q\right)}_{T/T_{l}}-S{\left(q\right)}_{T/\;T_{l}=1.02}$ at various levels of superheating (Figure S3). For eutectic Ti_27_Cu_73_, Δ*S*(*q*) shows a pronounced negative deviation in the *q*range of 4.8–5.2 Å^−^^1^ containing the second peak and its high‐*q* shoulder (Figure S3b), indicating that heating markedly increases topological disorder. For congruent Ti_50_Cu_50_, by contrast, the amplitude variation is much smaller and the shoulder is nearly invariant (Figure S3d); notably, the shoulder persists even at *T*/ *T_l_* = 1.08. The Ti_50_Cu_50_ melt is thus structurally far more robust against temperature change, which we attribute to stronger cluster correlations, as is evident in the 3‐D atomic configurations from our EM‐RMC modeling. The PDF data were used as experimental inputs (or structural constraints) in the EM‐RMC program to obtain three‐dimensional atomic configurations and cluster information of the alloy melts, as typically shown in Figure S4 and Figure 1g. By imposing both structural and energetic constraints, EM‐RMC ensures that the reconstructed configuration achieves a satisfactorily small discrepancy (χ^2^) between the simulated and experimental PDFs, as depicted in Figure 1e,f, while maintaining a relatively low potential energy. As such, EM‐RMC yields local atomic structures that are more accurate and physically realistic than those obtained from traditional RMC simulations (*see* Methods).

We then employed the Voronoi tessellation method to analyze atomic clusters of the alloy melts from the EM‐RMC reconstructed configurations [41, 42, 43]. This method constructs perpendicular bisector planes at the midpoints of the lines connecting a central atom to its neighbors, thereby partitioning the space into a set of Voronoi polyhedra that are densely packed around each atomic site. The atomic cluster types are described by the Voronoi index 〈*n*_3_,*n*_4_,*n*_5_,*n*_6_〉, where *n_k_* denotes the number of *k*‐sided polygons in a Voronoi polyhedron. For instance, the 〈0, 0, 12, 0〉 cluster corresponds to the icosahedral short‐range order (ISRO). To quantify the fivefold local symmetry, we introduced an order parameter $f_{i}^{5}$ for a type‐*i* cluster [44].

(2) $$f_{i}^{5}=\frac{n_{5}}{\sum_{k=3}^{6}n_{k}}$$

Essentially, $f_{i}^{5}$ is the proportion of pentagons in the type‐*i* Voronoi polyhedron; a larger $f_{i}^{5}$ signifies stronger local fivefold symmetry and a more icosahedral character. Following Wang et al. [44, 45, 46, 47, 48], we then calculated the average fivefold local symmetry parameter *W* by statistically averaging $f_{i}^{5}$ over all cluster types in a configuration

(3) $$W=\sum_{i}\left(f_{i}^{5}\times p_{i}\right)$$

where *p_i_* is the fraction of type‐*i* polyhedra among all Voronoi polyhedra. A higher *W* reflects a larger proportion of fivefold‐symmetric clusters, such as icosahedral and icosahedral‐like types. Figure 1h,i shows that with increasing temperature, both *W* and the fraction of the 〈0, 0, 12, 0〉 cluster (*f*_〈0,0, 12, 0〉_) decrease slightly for the Ti_27_Cu_73_ and Ti_50_Cu_50_ melts, respectively. Yet across the entire temperature range, Ti_27_Cu_73_ consistently exhibits higher *W* and *f*_〈0,0, 12, 0〉_ than Ti_50_Cu_50_, indicating that the eutectic melt possesses a greater fraction of fivefold‐symmetric clusters resembling ISRO. ISRO is incompatible with crystal lattices and is thought to inhibit nucleation, thus correlating positively with GFA [49, 50, 51, 52]. However, our observation that the congruent‐melting alloy (with lower ISRO) exhibits greater GFA contradicts this view, indicating that GFA in Ti‐Cu is not solely dictated by icosahedral cluster abundance.

### 2.2 Cluster Diversity

Based on the melt structure analysis above, we employed two parameters—the variance of cluster fraction distribution (σ^2^) and the Shannon entropy (*S*_sh_)—to characterize cluster diversity and configurational entropy. The former is defined as

(4) $$\sigma^{2}=\frac{\sum_{i=1}^{N}{\left(X_{i}-\mu \right)}^{2}}{N}$$

where *X_i_* is the fraction of cluster type *i*, μ is the mean fraction over all cluster types, and *N* is the total number of cluster types. A smaller σ^2^ indicates that the various cluster types have comparable fractions—a more uniform distribution—which is thought to hinder crystal nucleation and enhance GFA, as demonstrated by Wang and Flores et al. [35]. Complementarily, the Shannon entropy (*S*_sh_) measures local structural diversity.

(5) $$S_{\mathrm{sh}}=-\sum_{i=1}^{N}X_{i}\ln X_{i}$$

A larger *S*_sh_ corresponds to higher diversity and a more even distribution of local atomic environments, whereas a smaller *S*_sh_ indicates dominance by a few types of clusters [53, 54, 55, 56]. Figure 2a presents the temperature evolution of σ^2^ and *S*_sh_ for the two alloy melts. Over the entire temperature range, Ti_27_Cu_73_ exhibits larger σ^2^ (i.e., less uniform cluster distribution) but smaller *S*_sh_ (i.e., lower diversity) than Ti_50_Cu_50_. In other words, the congruent‐melting alloy Ti_50_Cu_50_ possesses both a more uniform cluster fraction distribution and a higher degree of structural diversity—precisely the characteristics expected for greater GFA. This is consistent with our experimental finding that Ti_50_Cu_50_ has higher GFA than the eutectic alloy. Moreover, σ^2^ and *S*_sh_ exhibit a clear negative correlation (Figure S5): σ^2^ ≈ −*aS*_sh_ + *b*, where *a* and *b* are constants. This relationship confirms that both parameters capture the same underlying feature—the configurational entropy arising from the distribution of cluster fractions.

**FIGURE 2:**
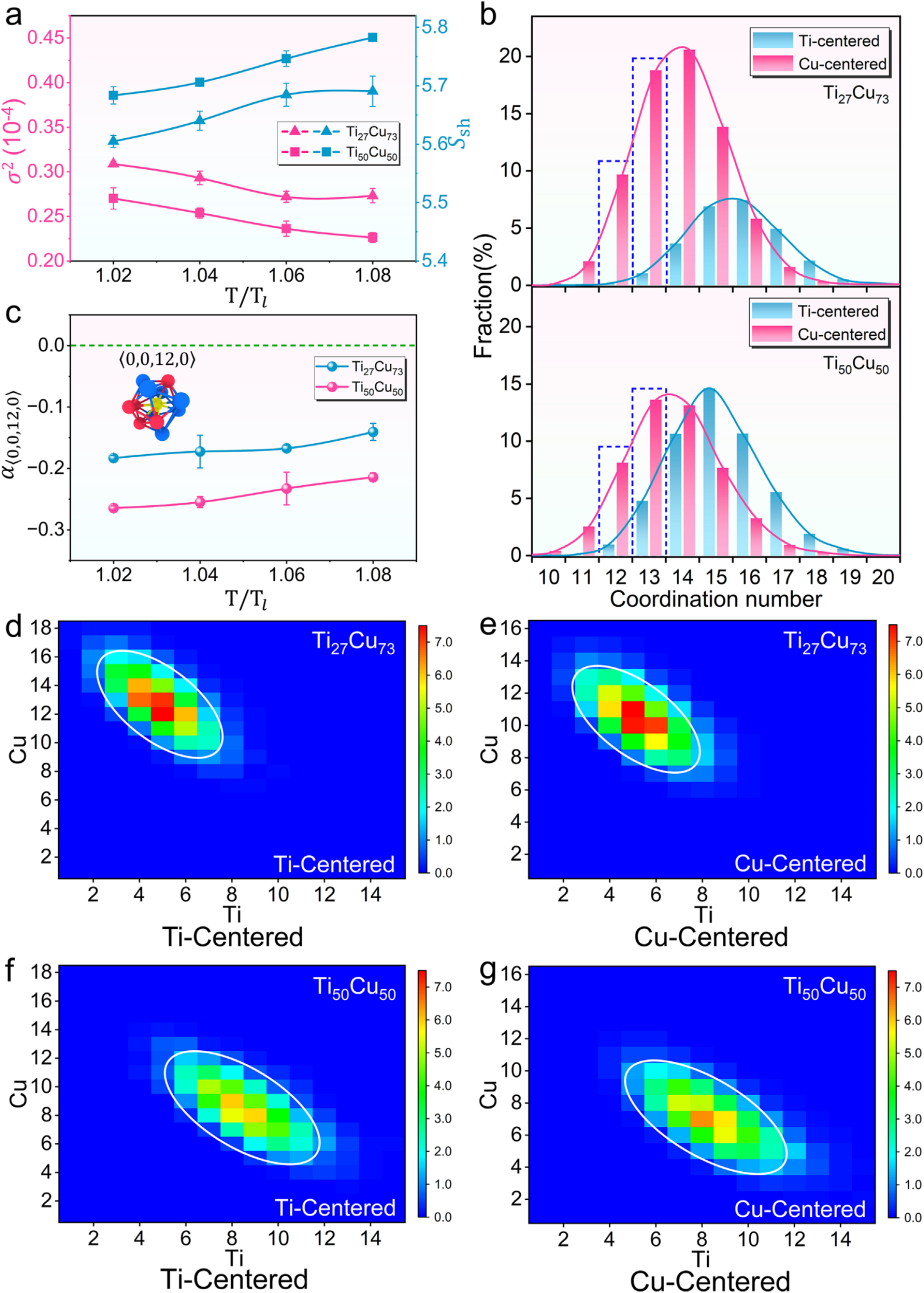
Cluster diversity in Ti_27_Cu_73_ and Ti_50_Cu_50_ melts. (a) Temperature dependence of cluster variance (σ^2^) and Shannon entropy (*S_sh_*). (b) Coordination number distributions with respect to Ti‐ and Cu‐centered clusters at *T*/ *T_l_* =  1.02 in Ti_27_Cu_73_ (top) and Ti_50_Cu_50_ (bottom). (c) Temperature dependence of Warren–Cowley parameter (α_motif_) for 〈0, 0, 12, 0〉 clusters. (d) and (e) 2‐D contour plots showing the fractions of Ti‐ and Cu‐centered clusters, respectively, in Ti_27_Cu_73_ at *T*/ *T_l_* =  1.02 as functions of partial coordination numbers of Cu (*z*_Cu_) and Ti (*z*_Ti_). (f) and (g) 2‐D contour plots showing the fractions of Ti‐ and Cu‐centered clusters, respectively, in Ti_50_Cu_50_ at *T*/ *T_l_* =  1.02 as functions of *z*_Cu_ and *z*_Ti_.

To understand why Ti_50_Cu_50_ has higher cluster diversity, we first analyzed the total coordination number (CN) distributions centered on different atomic types in the high‐temperature melts at *T*/ *T_l_* =  1.02. As shown in Figure 2b, the higher Ti content in Ti_50_Cu_50_ significantly enriches the diversity of clusters with CN values between 12 and 14. Based on the σ^2^ analysis, we decomposed the CN distribution into Ti‐centered and Cu‐centered clusters to identify the dominant types. The numbers of Ti and Cu atoms around a central atom are denoted by (*z*_Ti_,*z*_Cu_), with total CN *z*  = *z*_Ti_  + *z*_Cu_. Figure 2d,e shows that in Ti_27_Cu_73_, the (5, 12) of Ti‐centered cluster types and (5, 10) of Cu‐centered cluster types are overwhelmingly dominant, whereas Ti_50_Cu_50_ exhibits no such dominant type and has a much broader CN distribution (Figure 2f,g). Thus, the diversity of coordination environments changes significantly with alloy composition. We then examined the chemical short‐range order (CSRO) of 〈0, 0, 12, 0〉 clusters using the Warren–Cowley parameter  $\alpha_{\mathrm{motif}}=\frac{z_{\mathrm{Cu}}}{c_{\mathrm{Cu}}z}\;-1$, where *z*_Cu_ is the average coordination number of Cu atoms, *c*_Cu_ is the atomic fraction of Cu, and *z* is the total coordination number [57]. Figure 2c shows the temperature dependence of α_motif_ for the two melts. The deviation of α_motif_ from the random distribution (green dashed line) is larger for Ti_50_Cu_50_, indicating higher chemical order. Compared with Ti_27_Cu_73_, the increased Ti content promotes more unlike atomic bonds (Ti─Cu), thereby enriching cluster diversity.

### 2.3 Cluster Correlations

The local structure of a HTM is determined not only by the short‐range coordination environment (3–5 Å) but also by the topological connections between clusters at medium range (5–15 Å)—namely, cluster correlations. To quantify these correlations, we first classified clusters into ten groups based on their *f* ^5^ values by an interval of 0.1 using Equation (2). We then calculated the nearest‐neighbor correlation index *C_ij_* [28, 45, 58]:

(6) $$C_{ij}=\frac{P_{ij}}{P_{ij}^{*}}-1$$

where *P_ij_* is the probability that two polyhedron types (i, j) are nearest neighbors in a configuration, and $P_{ij}^{*}$ is the corresponding probability under a random distribution. Figure 3a,b presents the correlation statistics for the Ti‐Cu alloy melts at *T*/ *T_l_* =  1.02. Clusters with *f* ^5^ ≥ 0.6 or *f* ^5^ ≤ 0.3 tend to connect with their own kind, whereas those with 0.4 ≤ *f* ^5^ ≤ 0.6 repel each other. In other words, similar clusters preferentially interconnect, while dissimilar clusters avoid each other. Comparing the two alloy melts, Ti_50_Cu_50_ exhibits a significantly stronger tendency for high‐*f* ^5^ clusters to associate than Ti_27_Cu_73_, indicating that high fivefold‐symmetric clusters in Ti_50_Cu_50_ prefer face‐sharing connections.

**FIGURE 3:**
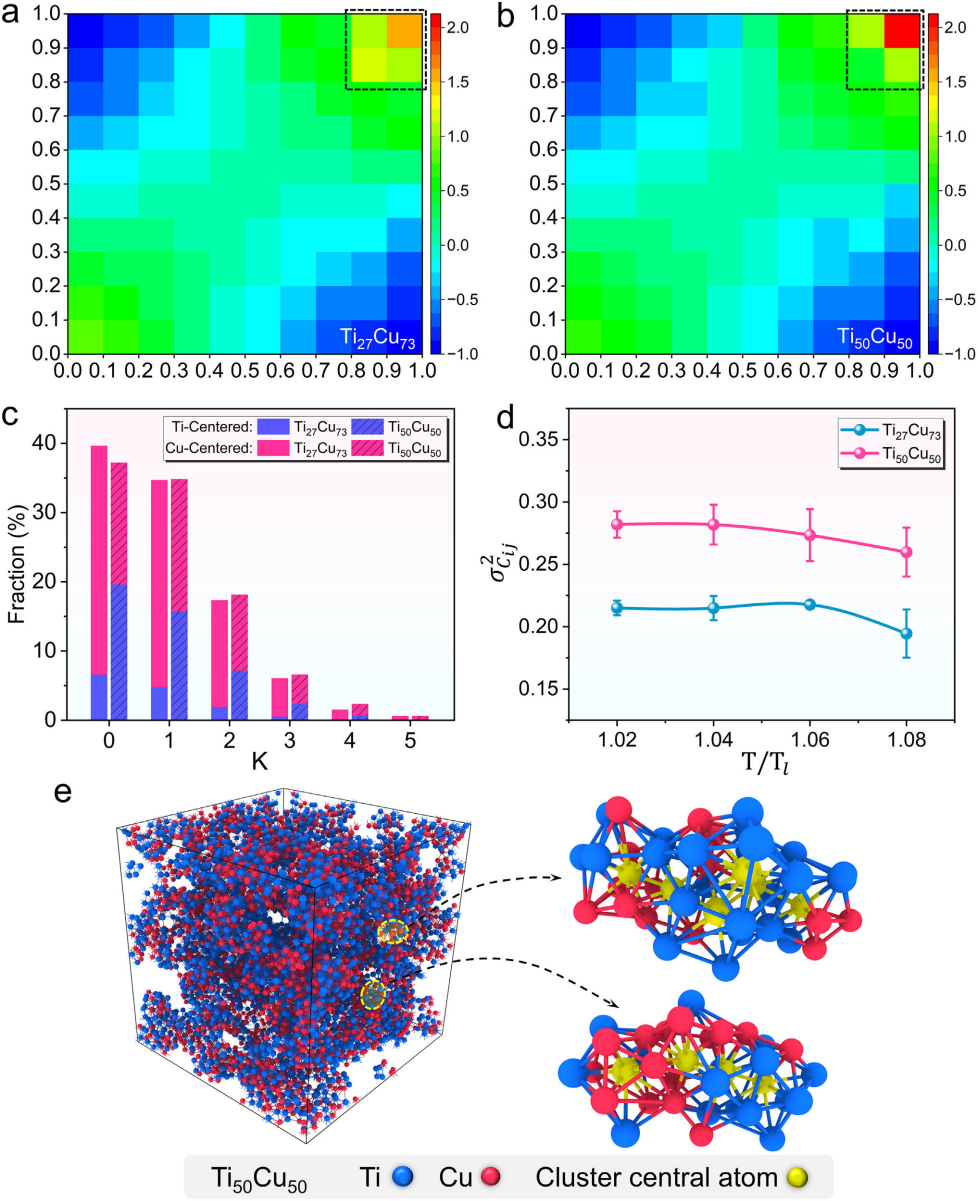
Cluster correlations in Ti‐Cu melts. (a,b) show the correlation index (*C_ij_*) between two cluster groups, each classified by its value of *f* ^5^at *T*/ *T_l_* =  1.02, respectively. Positive *C_ij_* indicates preferential connections between clusters; negative *C_ij_* points to mutual avoidance between clusters. The dashed boxes indicate strong correlations among high‐*f* ^5^ clusters. (c) Comparison of node degree (K) distributions for both 〈0, 0, 12, 0〉, 〈0, 1, 10, 2〉, and 〈0, 1, 10, 3〉 clusters (centered on Ti and Cu, respectively) in the Ti_27_Cu_73_ and Ti_50_Cu_50_ melts at *T*/ *T_l_* =  1.02. (d) Temperature dependence of cluster correlation variance ($\sigma_{C_{ij}}^{2}$) in Ti_27_Cu_73_ and Ti_50_Cu_50_ melts. (e) Representative three‐dimensional network of highly fivefold‐symmetric clusters in Ti_50_Cu_50_ at *T*/ *T_l_* =  1.02, together with enlarged views of typical connected motifs. Ti, Cu, and cluster central atoms are shown in blue, red, and yellow, respectively.

To further characterize the connectivity of clusters, we employed the concept of node degree based on the graph theory, as originally proposed by Wu et al. [59, 60]. In this scheme, a node is defined as the central atom of a cluster with a specific type (e.g., 〈0, 0, 12, 0〉), and two nodes are considered connected if they are nearest neighbors. This allows us to identify all connected clusters of the same type in a disordered system. The degree of a node (K) can then be defined as the number of neighboring atoms that serve as central atoms of the same cluster type, reflecting the local connectivity of that atom. Here, we extend this concept by allowing a node to represent the central atom of mixed cluster types, specifically high fivefold‐symmetric clusters such as 〈0, 0, 12, 0〉, 〈0, 1, 10, 2〉, and 〈0, 1, 10, 3〉. Figure 3c shows the distribution of *K* for the  〈0, 0, 12, 0〉, 〈0, 1, 10, 2〉, and 〈0, 1, 10, 3〉 clusters at *T*/ *T_l_* =  1.02. One can see that the increased Ti content in Ti_50_Cu_50_ significantly raises the proportion of connections with *K*  =  1 and 2, indicating that unlike atomic bonding not only enriches cluster types and connectivity but also provides an energetic basis for stabilizing the melt structure. As shown in Figure 3e, the larger fraction of Ti‐centered clusters expands the topological connections of high‐*f* ^5^ clusters and promotes the formation of a cluster network backbone that tends to resist crystallization [28, 59].

Furthermore, following the definition of σ^2^, we propose the cluster correlation variance ($\sigma_{C_{ij}}^{2}$) as a new metric to quantify correlation strengths among different cluster types, i.e.,

(7) $$\sigma_{C_{ij}}^{2}=\frac{\sum_{n}{\left(\varphi_{n}-\bar{c}\right)}^{2}}{n}$$

where φ_*n*_ is the correlation strength (*C_ij_*) between cluster types *i* and *j*, $\bar{c}$ is the mean of all *C_ij_* values, and *n* is the total number of *C_ij_* values counted. A larger $\sigma_{C_{ij}}^{2}$ indicates a stronger tendency for selective connections among one or several cluster types in the configuration, i.e., more pronounced cluster aggregation; conversely, a smaller value indicates no obvious preferential connectivity. This is exactly what is shown in Figure 3d, where the $\sigma_{C_{ij}}^{2}$ of Ti_50_Cu_50_ is consistently higher than that of Ti_27_Cu_73_ and is indicative of much stronger cluster correlations. In addition, $\sigma_{C_{ij}}^{2}$ appears insensitive to temperature, indicating that the high‐*f* ^5^ clusters in Ti_50_Cu_50_ maintain relatively strong correlations and resist disruption by thermal fluctuations. This provides a structural basis for the melt to withstand energy fluctuation‐induced cluster dissociation during cooling [61, 62].

### 2.4 Cluster Inheritance and Transformation

As mentioned earlier, we also collected ex situ synchrotron diffraction data from the MG ribbons of both alloys at room temperature and reconstructed their atomic configurations using the EM‐RMC method (Figure S6). A direct comparison between the glass and melt structures reveals significant differences, indicating that cluster inheritance and transformation occur during the glass transition—a point we will discuss in detail below.

Figure 4a,b shows the top 20 most abundant Voronoi polyhedra in the two alloy melts and their corresponding MGs, sorted by increasing *f* ^5^. Compared to the melts, the MGs have fewer clusters with 0.3 < *f* ^5^ ≤ 0.6 but a 2.2–2.5‐fold increase in high fivefold‐symmetric clusters (0.6 < *f* ^5^ ≤ 1). Notably, the melts contain 1100–1400 distinct cluster types, whereas the MGs contain only 600–800—a reduction of nearly half. Classifying the disappearing clusters by *f* ^5^ (Figure 4c,d) shows that for Ti_50_Cu_50_, 90.2% of the vanished clusters have *f* ^5^ ≤ 0.5, compared to 88.2% for Ti_27_Cu_73_. Thus, in Ti_50_Cu_50_, low‐*f* ^5^ clusters are preferentially transformed, while high‐*f* ^5^ clusters are largely retained and inherited into the glass. As a result, its high fivefold‐symmetric clusters keep significantly stronger correlations than in Ti_27_Cu_73_, consistent with the melt behavior, as shown by the *C_ij_* distributions of different clusters in Figure 4e,f. Thus, stronger inter‐cluster correlations survive cooling and are inherited, leading to greater structural mismatch with crystalline phases and a higher nucleation barrier in Ti_50_Cu_50_ [52]. On the other hand, Figure 4g presents the distribution of the node degree *K* for  〈0, 0, 12, 0〉, 〈0, 1, 10, 2〉, and 〈0, 1, 10, 3〉 clusters in the two MGs. Compared to the melt, the clusters with *K* = 2, 3 become more abundant (Figure S7), indicating significantly strengthened inter‐cluster correlations in the Ti_50_Cu_50_ glass. These atomic connection patterns, newly formed via cluster transformations during melt cooling, are entirely incompatible with crystalline structures. Consequently, they render the melt kinetically more rigid and increase its resistance to structural rearrangement (crystallization)—a key factor contributing to its greater GFA.

**FIGURE 4:**
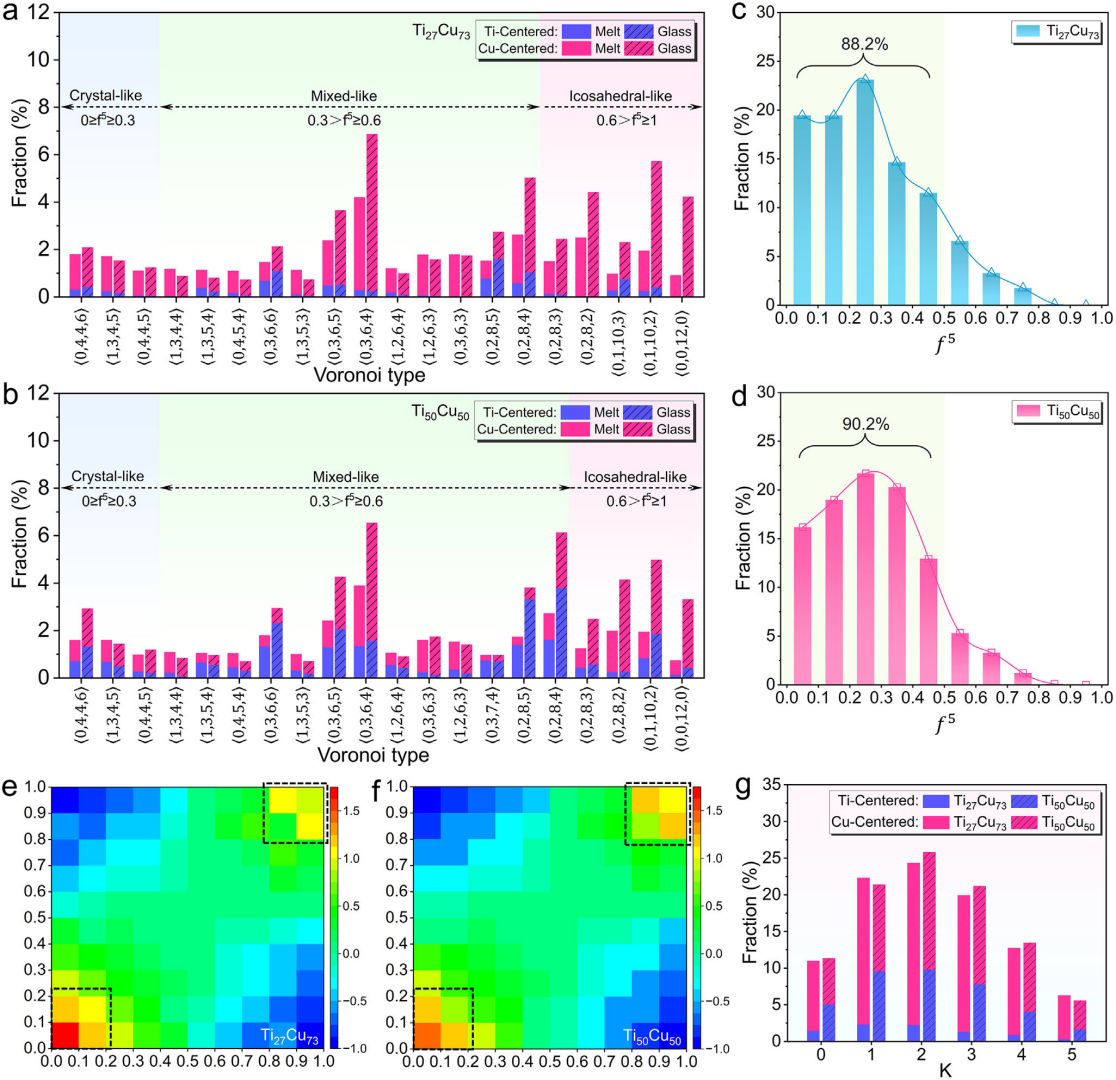
Cluster inheritance and transformation from melt to glass. (a,b) show the fractions of the top 20 Voronoi clusters (arranged in ascending order of f^5^) present in the Ti_27_Cu_73_ and Ti_50_Cu_50_ melts at T/ T_l_ =  1.02, respectively, together with those of their corresponding glasses at room temperature. (c) and (d) Distributions of Voronoi clusters that vanish across the glass transition as a function of f^5^ in Ti_27_Cu_73_ and Ti_50_Cu_50_, respectively. (e,f) Correlation index (C_ij_) between different cluster groups (classified by f^5^) in the Ti_27_Cu_73_ and Ti_50_Cu_50_ glasses, respectively. (g) Node degree (K) distributions for both 〈0, 0, 12, 0〉, 〈0, 1, 10, 2〉, and 〈0, 1, 10, 3〉 clusters (centered on Ti and Cu, respectively) in the Ti_27_Cu_73_ and Ti_50_Cu_50_ glasses.

Building on the above evidence, we further analyzed the atomic‐scale mechanisms of cluster transformations during the glass transition. As noted earlier, a Voronoi cluster is defined by the coordination polyhedron around its central atom—specifically, the numbers of faces, edges, and their spatial arrangement. Thus, mutual transformations between cluster types arise from thermally activated atomic motion that alters the local coordination topology, manifesting as changes in polygon side numbers, their disappearance, or creation [52, 62, 63]. As an example, Figure 5 illustrates the transformation pathways from three typical icosahedral‐like clusters to the ideal icosahedral cluster, as identified in the atomic configurations of the Ti_50_Cu_50_ melt and glass. The numbers on purple atoms indicate the side count of the perpendicular bisector polygon formed with the central atom. Two main pathways are identified. The first is topological distortion without a change in neighbor number (Figure 5a–d): atoms originally forming quadrilaterals and hexagons undergo vertical displacements, evolving into pentagons. Specifically, two yellow atoms move relative to each other, converting a quadrilateral into a pentagon and transforming a 〈0, 2, 8, 2〉 cluster into 〈0, 0, 12, 0〉. The second pathway is coordination reconstruction involving the gain or loss of neighbors, which occurs in two forms. One form (Figure 5b–d) involves an atom forming a triangle, converting 〈1, 0, 9, 3〉 to 〈0, 0, 12, 0〉. The other form (Figure 5c,d) involves a change in a quadrilateral, converting 〈0, 1, 10, 2〉 to 〈0, 0, 12, 0〉. Consequently, these transformation pathways reveal the atomic‐scale mechanism by which low‐fivefold‐symmetric clusters selectively convert to high fivefold‐symmetric clusters during the glass transition—a process that underpins the structural inheritance and evolution from melt to glass and provides a physical basis for understanding the anomalous GFA.

**FIGURE 5:**
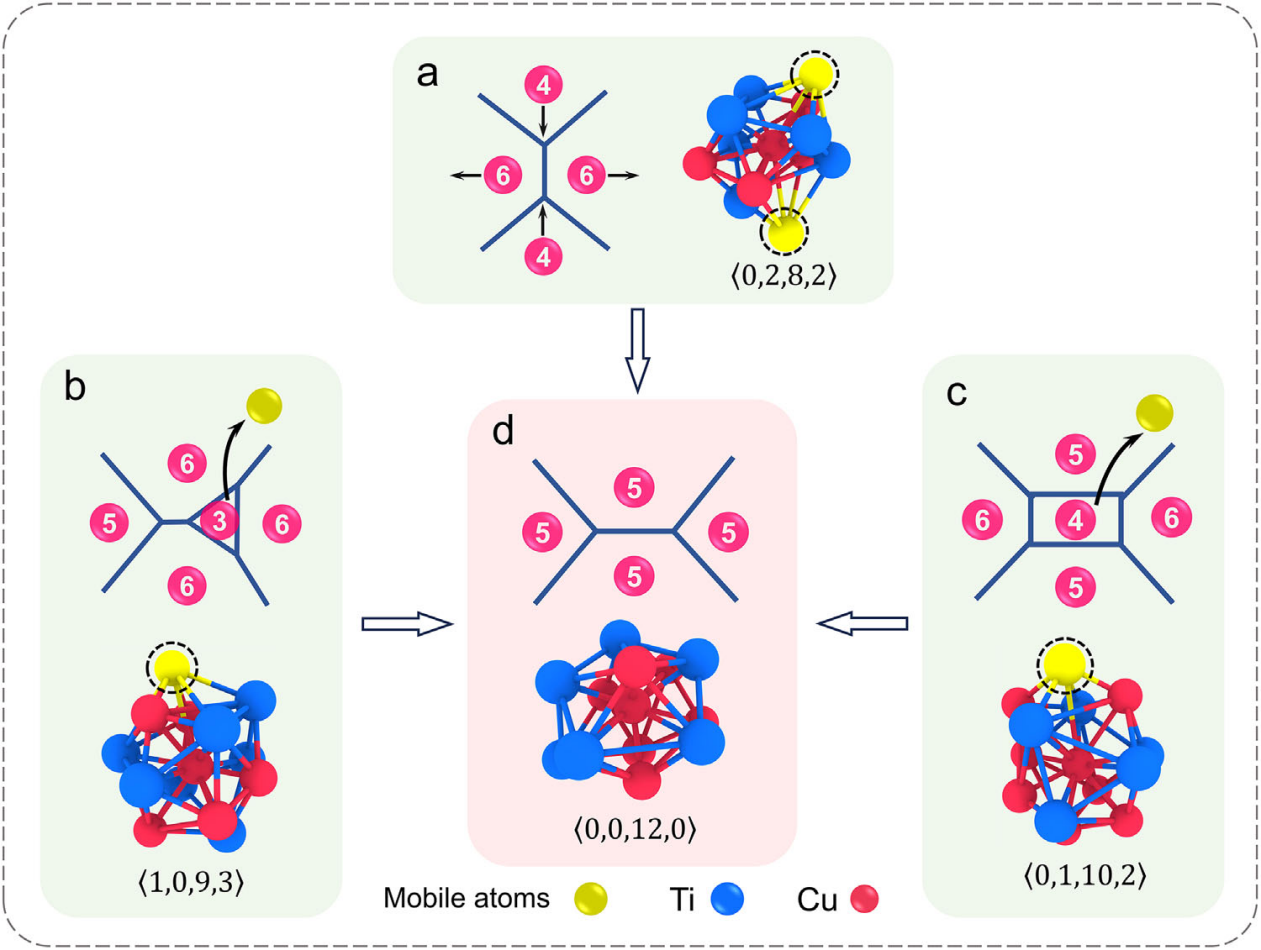
Mechanism of cluster transformation across the glass transition. Schematic illustrations and three‐dimensional atomic configurations showing the transformation of three representative icosahedron‐like clusters ((a) 〈0, 2, 8, 2〉, (b) 〈1, 0, 9, 3〉, and (c) 〈0, 1, 10, 2〉) into an icosahedral cluster ((d) 〈0, 0, 12, 0〉), respectively. The number in each purple circle indicates the edge number of the polygon formed on the perpendicular bisector plane between the labelled atom and the cluster central atom. Yellow balls mark those atoms that shift (a) or move away (b, c) during the transformation. Blue and red balls represent original Ti and Cu atoms, respectively.

## 3 Discussion

The observation that a congruent‐melting alloy (Ti_50_Cu_50_) outperforms its eutectic neighbor (Ti_27_Cu_73_) in glass‐forming ability directly contradicts the decades‐old eutectic rule and the classical nucleation‐based *T_rg_* criterion. While previous attempts to explain such an anomaly invoked metastable phase diagrams or eutectic coupled zones [20], they could not account for why the greater GFA sometimes appears at equiatomic or congruent compositions. Our work provides a fundamentally different answer: the origin lies not in the competition among crystalline phases, but in the *structure and stability of the melt itself*—the direct precursor to the glass.

Using in situ synchrotron diffraction and EM‑RMC reconstruction, we reveal that the congruent melt is not simpler but structurally more complex. This complexity manifests in three interrelated aspects: higher cluster diversity, stronger inter‑cluster correlations, and more effective cluster transformations during cooling—all of which contribute to its greater GFA, as elaborated below.

First, cluster diversity acts as an entropy‐driven barrier. Ti_50_Cu_50_ exhibits higher cluster diversity than Ti_27_Cu_73_, as evidenced by its smaller σ^2^ and larger *S*_sh_ (Figures 6a and 2a). A smaller σ^2^ indicates that the populations of different cluster types are nearly uniform, so that many distinct local motifs coexist in similar proportions, thus yielding a highly disordered structure as no single cluster type dominates. In parallel, the larger *S*_sh_ reflects a broad and balanced distribution of local atomic environments, indicating the absence of any dominant structural motif. Together, these complementary metrics demonstrate that the high‐temperature Ti_50_Cu_50_ melt possesses much richer cluster diversity than the eutectic Ti_27_Cu_73_ melt. This diversity originates from the higher configurational entropy (*S*_conf_) of the equiatomic composition. *S*_conf_ can be evaluated using the ideal solution approximation  *S*_conf_ =   − *R*[*x*_Ti_ln *x*_Ti_ + *x*_Cu_ln *x*_Cu_], which is maximal at the equiatomic composition Ti_50_Cu_50_ (0.693R) and lower at the eutectic composition Ti_27_Cu_73_ (0.583R). The higher Shannon entropy and lower cluster variance observed for Ti_50_Cu_50_ provide structural fingerprints consistent with this larger configurational diversity. Figure 6d shows that, both before and after the glass transition, the fraction of Ti─Cu bonds in Ti_50_Cu_50_ consistently exceeds the random‐bonding reference, indicating strong unlike atomic bonding—consistent with the negative mixing enthalpy of Ti‐Cu (−9 kJ/mol). Consequently, Ti_50_Cu_50_ possesses a much richer variety of Ti‐centered clusters (Figure 2b). The coexistence of many distinct cluster types—a “disordered structure”—creates geometric frustration that raises the structural barrier for nucleation [35]. In contrast, the eutectic alloy, with fewer unlike atomic bonds, suffers from limited cluster diversity, especially among Ti‐centered types (Figures 2b and 3c). Furthermore, *S*_sh_ is a direct proxy for *S*_conf_; both decrease with temperature and reach their minima in the glass (Figure 6a). According to Adam‐Gibbs theory [29], higher *S*_conf_ (and thus higher *S*_sh_) leads to higher viscosity and lower diffusivity. Therefore, cluster diversity acts as an entropic barrier, kinetically hindering nucleation and thereby enhancing GFA.

**FIGURE 6:**
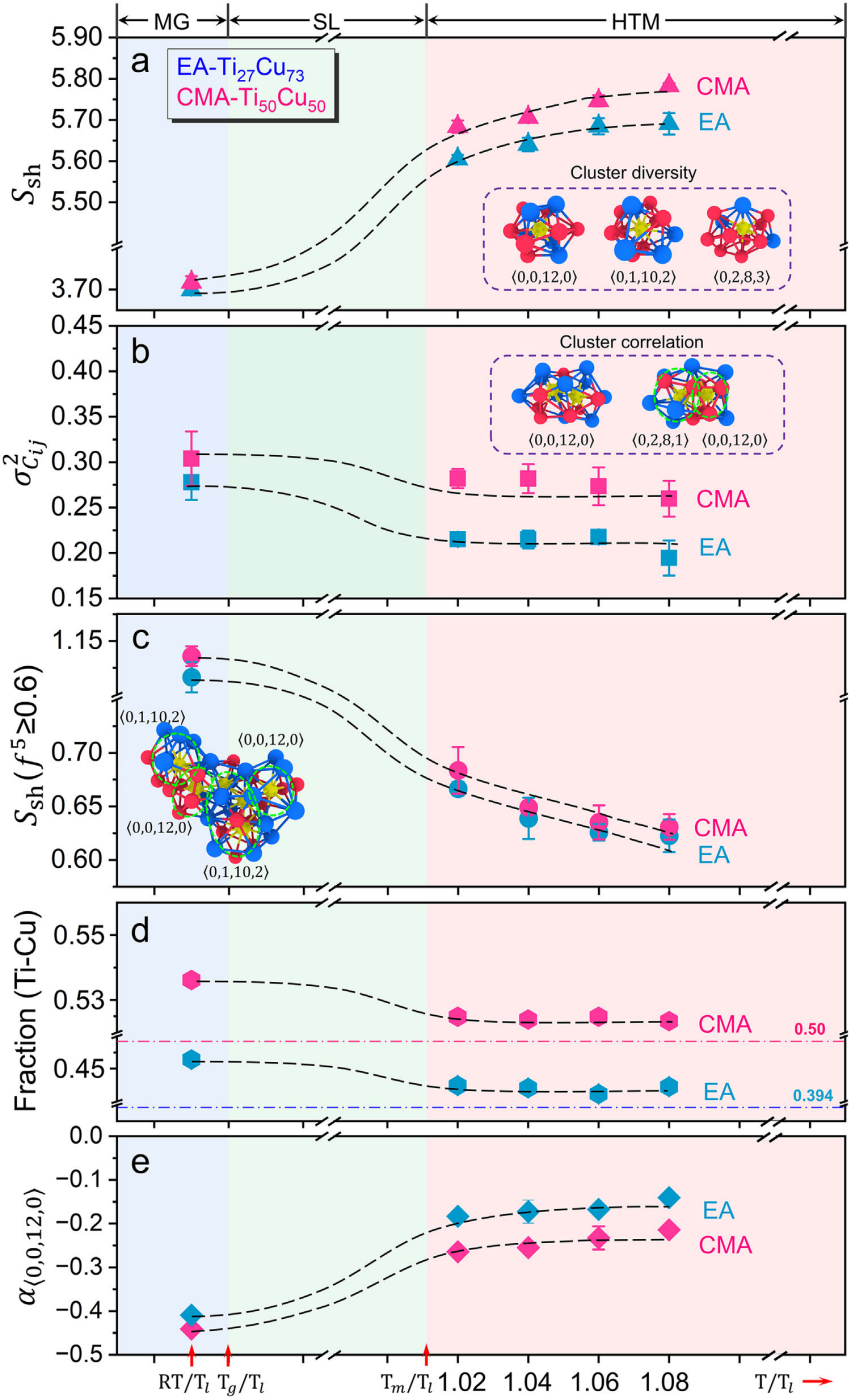
Structural evolution from melt to glass. The panels show the temperature dependence of (a) overall Shannon entropy (*S*_sh_)−indicative of cluster diversity; (b) cluster correlation variance ($\sigma_{C_{ij}}^{2}$)−indicative of cluster correlations; (c) *S*_sh_ for high fivefold‐symmetric clusters (*f* ^5^ ≥ 0.6); (d) Ti─Cu bond fraction, and (e) Warren–Cowley parameter (α_motif_) of 〈0, 0, 12, 0〉. The dashed lines are guides to the eye. Blue, green, and red backgrounds denote the temperature regions corresponding to metallic glass (MG), supercooled liquid (SL), and high‐temperature melt (HTM) regions, respectively.

Second, inter‐cluster correlations serve as an enthalpy‐driven stabilizer. Ti_50_Cu_50_ also exhibits stronger inter‐cluster correlations, reflected in its larger $\sigma_{C_{ij}}^{2}$ (Figure 6b) and higher populations of locally connected clusters with node degrees *K*  =  1, 2 (Figure 3c). Unlike atomic bonding not only promotes cluster diversity but also provides the structural basis for strong correlations among high fivefold‐symmetric clusters. As shown in Figure 6e, icosahedral short‐range order in Ti_50_Cu_50_ is chemically more ordered than in Ti_27_Cu_73_, indicating that Ti─Cu bonds facilitate the topological linking of high‐*f* ^5^ clusters. Although this ordering partially reduces configurational entropy, it lowers the system's enthalpy and thereby enhances melt stability. The resulting locally correlated structures suppress single‐atom diffusion while increasing the characteristic size of cooperatively rearranging regions (CRRs) [28, 29]. Relaxation therefore becomes increasingly collective, occurring through the cooperative rearrangement of atomic groups or motifs. Furthermore, the strong correlations among high‐*f* ^5^ clusters build a cluster network backbone (Figure 3e) that extends the geometric frustration initiated by cluster diversity. Hence, inter‐cluster correlations act as an enthalpy‐driven barrier: they raise the energetic cost for atomic rearrangement and reinforce structural resistance to nucleation—both of which are favorable for glass formation.

Third, selective cluster transformation bridges diversity and correlation. During cooling, Ti_50_Cu_50_ exhibits a higher degree of cluster transformation than Ti_27_Cu_73_ (Figure 4a–d). As temperature decreases, the Shannon entropy of clusters with *f* ^5^ ≥ 0.6 increases (Figure 6c), revealing preferential retention and selective conversion toward high fivefold‐symmetric clusters. Contrary to the traditional view of a glass as a “frozen liquid,” our results show that the melt actively restructures during undercooling: low‐*f* ^5^ clusters—which are prone to crystallization—are converted into high‐*f* ^5^ clusters that possess strong geometric frustration. This is an intrinsic response of the melt to avoid ordering. Ti_50_Cu_50_, with its abundant unlike atomic bonding and high cluster diversity, is particularly capable of driving this conversion. The newly formed high‐*f* ^5^ clusters serve as bridges between diversity and correlation, promoting the formation of strongly correlated local structures that are the building blocks of cooperatively rearranging regions (CRRs). Because fivefold symmetry is non‐crystallographic and incompatible with periodic translation, it cannot form a crystalline lattice. Consequently, the high convertibility and strong correlations in Ti_50_Cu_50_ directly impede crystal embryo formation, making selective transformation a key factor for the anomalous GFA.

Overall, the complex melt structure of the congruent alloy arises from the synergy between entropy‐driven cluster diversity (enabled by high configurational entropy) and enthalpy‐driven inter‐cluster correlations (enabled by unlike atomic bonding). Diversity creates geometric frustration; correlations stabilize the network and raise energetic barriers; and selective transformation during cooling amplifies both effects. This interplay, i.e., entropy–enthalpy competition manifested through cluster diversity, correlation, and transformation, constitutes the physical origin of the anomalous GFA.

The generality of this mechanism is further supported by the Ni–Nb system, which provides a natural reverse test. Unlike Ti–Cu, where the superior glass former is the congruently melting composition, Ni–Nb exhibits the opposite behavior, in that the eutectic composition Ni_58.5_Nb_41.5_ is a significantly better glass former than the congruently melting Ni_75_Nb_25_, as established by the γ* criterion. If the traditional eutectic rule were the governing principle, this system would offer no further insight, as it would simply conform to expectation. Yet when the high‐temperature melts of these two compositions are compared using the same structural metrics applied to Ti–Cu, the eutectic melt again emerges as the structurally more complex one, with consistently higher Shannon entropy, lower cluster fraction variance, and stronger inter‐cluster correlations across the superheated regime examined (see Note S1 and Figures S8 and S9 for full details of the Ni–Nb calculations, including the temperature‐dependent evolution of all structural metrics). In other words, the composition that is the better glass former in each system is the one whose melt is structurally more complex, irrespective of whether that composition is eutectic or congruent. This strongly suggests that it is the diversity and spatial connectivity of local structural motifs in the melt, rather than proximity to a eutectic point, that ultimately governs the competition between glass formation and crystallization.

This mechanistic picture can be placed in a broader theoretical basis using the two‐order‐parameter framework of Tanaka et al. [64, 65, 66], which proposes that liquids possess two competing orderings. A density ordering drives crystallization by maximizing local density, while a bond ordering favors a local symmetry such as icosahedral order that is geometrically incompatible with the crystal. Vitrification arises from the frustration between the two, with a stronger bond‐ordering tendency corresponding to a better glass former and a less fragile liquid. This maps naturally onto our structural metrics, where Shannon entropy (*S*_sh_) and cluster fraction variance (σ^2^) quantify the richness of bond ordering, and the cluster correlation variance ($\sigma_{C_{ij}}^{2}$) measures the spatial connectivity of the resulting motifs. Our results are fully consistent with this picture. In both Ti–Cu and Ni–Nb, the superior glass former is the melt with greater bond‐ordering diversity and more extensive inter‐cluster correlations, while the poorer glass former adopts a simpler bond‐ordered structure that is more readily destabilized by the competing density ordering. Melt structural complexity thus provides a structural realization of the bond‐ordering parameter that Tanaka's framework identifies as the key control variable for glass formation.

These results have significant implications for metallic glass design. Beyond the traditional strategies of targeting deep eutectics or maximizing icosahedral order, one should also consider alloy compositions that promote structural complexity in the melt. Congruently melting intermetallic compounds, particularly those with high configurational entropy arising from chemical complexity on specific lattice sites, emerge as promising precursors for bulk metallic glasses, as these intermetallics present substantial barriers to crystallization, thereby favoring glass formation. These alloys are distinct from high‐entropy alloys of the Cantor type [67], which, as noted extensively in the literature, exhibit poor glass‐forming ability owing to the fast nucleation and growth kinetics of the single‐phase solid solution phase. This can be exemplified by the classic Zr‐Cu binary system, in which adding Al and/or Ni into Zr_50_Cu_50_ can significantly improve GFA, leading to bulk metallic glasses such as Zr‐Cu‐Al and Zr‐Cu‐Ni‐Al [68, 69, 70]. Because their DSC curves exhibit a single melting peak, these alloys have long been considered deep eutectic compositions. However, a single sharp melting peak is also a typical thermodynamic signature of a congruent‐melting alloy. This raises the possibility that the high GFA of many well‐established BMGs may originate not from eutectic effects but from the intrinsically high mixing entropy and complex melt structure. Thus, the metrics proposed here provide a new framework for engineering the liquid state by leveraging both the configurational entropy of mixing and the enthalpy of mixing among constituent elements, opening new avenues for designing high‐GFA alloys beyond traditional eutectic‐based guidelines.

## 4 Conclusions

This work resolves the paradoxical GFA behavior in the Ti‐Cu system by directly probing the atomic structure of high‐temperature melts—a perspective long neglected in favor of crystallization kinetics. Through a combination of in situ synchrotron diffraction, aerodynamic levitation, and our newly developed EM‐RMC method, we show that the congruent‐melting alloy Ti_50_Cu_50_, despite its higher melting point, forms a glass superior to the eutectic Ti_27_Cu_73_ because its melt is inherently more complex. Quantitatively, we introduced three metrics to characterize the melt and glass structures that go beyond conventional short‐range order analysis: cluster variance (σ^2^) and Shannon entropy (*S*_sh_) for entropy‐driven cluster diversity, and cluster correlation variance ($\sigma_{C_{ij}}^{2}$) for enthalpy‐driven inter‐cluster associations. The congruent melt exhibits both greater diversity and stronger correlations, arising from entropy–enthalpy competition. This structural complexity is not merely a static feature; it is inherited across the glass transition and even amplified via selective cluster transformations, creating a dual barrier to crystallization. As such, our findings reveal that melt structural complexity—rather than solely a specific eutectic composition or icosahedral order—plays a key role in governing GFA, as exemplified by the Ti–Cu system, and offer a new perspective for understanding the glass transition at the atomic cluster level. This insight offers a new strategy for glass design, shifting focus from the eutectic rule alone to the structural properties of alloy melts.

## 5 Methods

### 5.1 Materials Preparation

High‑purity Ti (≥99.99%) and Cu (≥99.99%) were used as raw materials. Ti_27_Cu_73_ and Ti_50_Cu_50_ alloy ingots were prepared by arc melting the constituent elements under an argon atmosphere. To ensure chemical homogeneity, each ingot was re‑melted four times by flipping front and back alternately. The homogenized ingots were ground and polished to remove surface oxides, then cut into small pieces. Each piece was then arc‑melted and cast into a spherical mold to produce a sphere approximately 2 mm in diameter, which was used for aerodynamic levitation experiments. A single‑roller melt‑spinning apparatus was used to produce MG ribbons of the two alloys, with typical dimensions of 3 mm in width and 25–35 µm in thickness. Since our structural complexity parameters are temperature‐dependent, the melt‐spinning temperature (*T_q_*) also warrants consideration. We therefore performed melt‐spinning experiments at *T_q_* = *T_l_* +100, 150, and 200 K, respectively. At all three temperatures, the products are consistently fully amorphous, provided the cooling rate (i.e., the rotation speed of the copper wheel) is kept constant and sufficiently high. This shows that within our accessible superheating window, *T_q_* alone does not determine whether the product is glassy or crystalline. The outcome is governed by the cooling rate. In principle, this is expected: above *T_l_*, the melt is ergodic and remains in internal equilibrium at *T_q_*. It therefore passes through the same temperature sequence upon cooling regardless of the starting temperature. Consistent with this, the difference in GFA of the two alloys manifests in their different critical cooling rates rather than in the ribbon products obtained under fixed, sufficiently fast quenching. As such, in the Results section, we present measurements on melt‐spun ribbons of the two alloys, quenched from *T_l_* +150K.

### 5.2 Aerodynamic Levitation

A custom‑built aerodynamic levitator was used to levitate each selected Ti‐Cu alloy sphere while laser‐heated to approximately 150°C above its melting temperature (*see* Supplementary Video), with concurrent monitoring by in situ high‐energy synchrotron x‐ray diffraction (Figure S1a). The experiments were conducted on the BL12SW beamline of the Shanghai Synchrotron Radiation Facility (SSRF), and the setup is schematically shown in Figure S10. The levitator comprises a 100 W CO_2_ laser, a CCD camera, an infrared pyrometer, and the furnace body. To achieve a sufficiently large scattering vector *q*, the furnace window was set to an angle of 100°, allowing *q* values above 20 Å^−^^1^. Our aerodynamic levitator is tightly sealed against air ingress and operates with high‐purity argon and a deoxidation unit; before each experiment, the chamber was evacuated below 10^−^^4^ Pa and backfilled with deoxygenated high‐purity argon to minimize oxygen contamination. Although oxides may form during prolonged isothermal holding [33], all structural data were acquired immediately upon complete melting and during continuous heating—a very short data‐acquisition window of approximately 5 s. Under these conditions, the sharp Bragg peaks of the initial crystalline phases vanish and evolve into broad diffuse halos (Figure S11a,c); the slight intensity fluctuations of the primary diffuse peak after melting are attributed to sample‐position fluctuations during aerodynamic levitation. Critically, the melt patterns (Figure S11b,d) show only liquid diffuse scattering, with no diffraction peaks corresponding to oxides, confirming no detectable oxidation during the data‐acquisition window.

### 5.3 High‐Energy Synchrotron X‐Ray Diffraction

A PILATUS3 X CdTe 2 M area detector was used in the above‐mentioned in situ synchrotron x‐ray diffraction experiments, with a wavelength of 0.1258 Å (energy 98.55 keV). The sample‐to‐detector distance was calibrated using a CeO_2_ standard. Data acquisition commenced simultaneously with laser heating, and each pattern was collected with an exposure time of 0.2 s. No oxide peaks were discernible in the collected patterns, indicating negligible or no oxidation during melting. The raw data were reduced using the GSAS‐II program to obtain the diffraction intensity *I*(*q*) as a function of *q* [71]. Subsequently, the PDFgetX2 program was employed for background subtraction, absorption correction, and normalization, yielding the structure factor *S*(*q*) and the reduced pair distribution function $G\left(r\right)=\frac{2}{\pi }\;\int_{0}^{\infty }q\left[S(q)-1\right]\sin\left(qr\right)\;dq$, where *r* is the interatomic distance [72]. As for the metallic glasses, diffraction data were collected ex situ from melt‐spun ribbons with an exposure time of 60 s each. The data reduction and processing procedure to obtain *S*(*q*) and *G*(*r*) is the same as described above.

### 5.4 Energy Minimization‐Enhanced Reverse Monte Carlo (EM‐RMC) Simulation

The measured synchrotron x‐ray PDF data (e.g., *G*(*r*)) were then used to reconstruct three‐dimensional atomic configurations of the HTMs and corresponding MGs for each alloy, as implemented in our newly developed energy minimization‐enhanced reverse Monte Carlo (EM‐RMC) simulation program. Conventionally, the RMC method is based on the Metropolis Monte Carlo algorithm and allows an initial structure (usually a random configuration) to be rearranged by randomly moving atoms one at a time to minimize the loss function χ^2^, *i.e*., the sum of squared differences between each simulated and experimental PDF data point [73],

(8) $$\chi^{2}=\sum_{i=1}^{n}{\left(G{\left(r\right)}_{sim,i}-G{\left(r\right)}_{exp,i}\right)}^{2}$$

where the subscripts “*sim*” and “*exp*” denote the simulated and experimental data, respectively, and *i* represents the *i*th data point of *G*(*r*). However, owing to the use of only one set of experimental PDF dataset as the constraint, this method has long been known to be unreliable, often yielding configurations with unrealistically high energies and more distorted local atomic arrangements [74]. To resolve this energetic dilemma, we introduce an additional energetic constraint into the conventional RMC procedure by imposing interatomic potentials that allow for the energy minimization (EM) after substantially reducing the χ^2^ value. We thus term this approach EM‑RMC, which enables the reconstruction of structurally reliable and energetically favorable configurations from experimental PDF data.

Figure S1b shows the flowchart of the EM‐RMC procedure. In each run, an initial configuration together with the experimental PDF data is first processed through the RMC module, in which a new configuration is generated by randomly moving atoms one at a time over *N* trial steps until a comparatively low χ^2^ is achieved. This configuration is then fed into the EM module, where all atoms are moved simultaneously over *M* times using a conjugate gradient method [75] to produce a new configuration with a lower potential energy (*E_sim_*). This EM‐enhanced configuration is subsequently returned to the RMC module, forming an iterative loop. This cycle is repeated until the cumulative number of RMC steps reaches a preset value.

Note that each EM‐RMC run requires *N* RMC steps and *M* EM steps in series to produce a configuration, which can be characterized by a control parameter δ  = (χ^2^ + Δ*E*/(*k_B_T*)) /2, where Δ*E*  = *E_sim_*  − *E_target_* is the difference in potential energy between the simulated configuration (*E_sim_*) and the target configuration (*E_target_*), *k_B_* is the Boltzmann constant, and *T* is the temperature. As will be shown below, δ is a manifestation of both structural and energetic constraints. To efficiently explore the combinatorial (*N*, *M*) space, parallel computations are employed to simultaneously generate *k_N_* × *k_M_* sets of configurations, where *k_N_* and *k_M_* represent the numbers of N and M values, respectively. Each such configuration has a corresponding δ value, i.e., δ (*N*, *M*), and an optimal configuration can then be obtained by selecting the one having the minimum δ value globally.

We then validate the EM‐RMC method by testing the reliability of the EM‐RMC reconstructed configurations using a *known* melt structure as the benchmark. Specifically, this known melt structure of Ti_50_Cu_50_ at 1296 K is generated a priori using molecular dynamics (MD) simulations, as implemented in the LAMMPS program [76], where interatomic interactions are described by a reliable Modified Embedded Atom Method (MEAM) potential [77]. We then calculate the total pair distribution function and the potential energy of this *known* structure, and use the former as a hypothetical experimental data input and the latter as *E_target_* in calculating the δ parameter in the EM‐RMC simulation. As such, this MD‐generated *known* structure is indeed the target configuration that the EM‐RMC strives to achieve. Following the EM‐RMC procedure, we start from an initially randomized configuration within a cubic simulation box of 6.9 × 6.9 × 6.9 nm^3^ that contains 20 000 atoms under periodic boundary conditions. The optimal configuration is obtained after 9 000 000 steps, resulting in a minimum δ value of 0.212 with χ^2^ = 0.43 and the reduced energy = −3.92 eV/atom. Similarly, we also conduct conventional RMC to generate a configuration after 5 000 000 steps, which corresponds to a χ^2^ value of 0.19. Consequently, the reliability of EM‐RMC and conventional RMC can be assessed by examining how well the partial PDFs (*g_ij_* (*r*)) of reconstructed configurations match those of the MD‐generated target configuration, respectively, as shown in Figures S12 and S13. Both conventional RMC and EM‐RMC successfully reproduce the total *G*(*r*) of the target configuration (Figure S12), yet their partial PDFs (*g_ij_* (*r*)) differ significantly. Conventional RMC produces substantially large deviations, including shifted and distorted peaks (Figure S13a,c), corresponding to an average R^2^ of 0.46. EM‐RMC, however, closely matches the target partial PDFs with negligible errors (Figure S13b,d), with an average R^2^ of 0.97. These results demonstrate that, by imposing both structural and energetic constraints, EM‐RMC is capable of producing atomic configurations with a satisfactorily smaller χ^2^ while maintaining a relatively low potential *E_sim_*, which are more accurate and physically realistic than those obtained by conventional RMC alone.

Accordingly, EM‐RMC was applied to reconstruct the melt and glass structures of Ti_27_Cu_73_ and Ti_50_Cu_50_ from the experimental PDFs measured by synchrotron x‐ray diffraction. For both alloys, initial atomic configurations were built via MD simulations in a 20 000‐atom cubic box with periodic boundary conditions. The respective box sizes were 6.7 × 6.7 × 6.7 nm^3^ and 6.9 × 6.9 × 6.9 nm^3^ for Ti_27_Cu_73_ and Ti_50_Cu_50_, respectively. For high‐temperature melt structures, starting from random atomic positions, each system was heated to the target temperature and held for 100 ps in the NPT ensemble at zero pressure, using a 2 fs time step. The resulting MD structure was refined with EM‐RMC until the δ value reached its minimum, as typically shown in Figure S14a,b for Ti_27_Cu_73_ and Ti_50_Cu_50_, respectively. The glass structure was reconstructed using the same methodology as described above, and the corresponding (N, M) screening results are presented in Figure S14c,d. All structural statistics reported in this work are derived from Voronoi tessellation using the full set of Voronoi faces for each atom, without imposing any distance, face‐area, or edge‐length cut‐off. Because the Voronoi construction is parameter‐free, the resulting polyhedron statistics are not subject to the arbitrariness associated with distance‐based neighbor definitions and are fully consistent across all compositions, temperatures, and states analyzed.

## Author Contributions

**Wei Chen**: writing – original draft, methodology, software, formal analysis, investigation, data curation, conceptualization**. Xuerui Wei**: methodology, software, investigation, validation. **Chengzhe Wang**: methodology, investigation. **Zhichao Lu**: resources, methodology, investigation, conceptualization. **Fan Zhang**: investigation. **Yan Huang**: investigation. **Yibo Zhang**: investigation. **Liang Wang**: investigation. **Saichao Cao**: resources, methodology. **Chunyin Zhou**: resources, methodology. **Ke Yang**: resources, methodology. **Zhendong Fu**: investigation. **Jinkui Zhao**: resources, methodology, funding acquisition. **Dongbai Sun**: resources, methodology. **Fanqiang Meng**: writing – review and editing, supervision, resources, methodology, conceptualization. **Zhou Zhou**: writing –review and editing, supervision, resources, methodology, conceptualization. **Xun‐li Wang**: resources, validation. **Weihua Wang**: resources, validation, methodology. **Dong Ma**: writing – review and editing, supervision, resources, methodology, funding acquisition, validation, conceptualization. **Wei Chen** and **Dong Ma** wrote the manuscript after discussions and with contributions from all the authors.

## Conflicts of Interest

The authors declare no conflicts of interest.

## Supporting information




**Supporting File 1**: advs78359‐sup‐0001‐SuppMat.docx.

**Supporting File 2**: advs78359‐sup‐0002‐VideoS1.mp4.

## Data Availability

The data that support the findings of this study are available from the corresponding author upon reasonable request.
