# Supplementary material for: To Be or Not to Be Eutectic: Anomalous Glass Forming Ability Governed by Melt Structural Complexity
Source: Adv Sci (Weinh). 2026 Oct 18:e78359. Online ahead of print. doi: 10.1002/advs.78359 (PMC13677046; doi:10.1002/advs.78359)
Supplement: Supplementary file 1 [file ADVS-9999-e78359-s002.docx]

Supporting Information

**To Be or Not to Be Eutectic: Anomalous Glass Forming Ability Governed by Melt Structural Complexity**

Wei Chen^1,2,3,4^, Xuerui Wei^3,4^, Chengzhe Wang^3,4,5^, Zhichao Lu^3,4^*, Fan Zhang^3,4^, Yan Huang^3,4^, Yibo Zhang^3,4,7^, Liang Wang^3,4^, Saichao Cao^6^, Chunyin Zhou^6^, Ke Yang^6^, Zhendong Fu^4^, Jinkui Zhao^4^, Dongbai Sun^8,9^, Fanqiang Meng^5^*, Zhou Zhou^1,2^*, Xun-li Wang^7^,Weihua Wang^3^, Dong Ma^3,4^*

^1^ Center for Advanced and Ultrafast Photonic Science, School of Physics, Sun Yat-sen University, Guangzhou 510275, China.

^2^ Center for Neutron Science and Technology, Guangdong Provincial Key Laboratory of Magnetoelectric Physics and Devices, School of Physics, Sun Yat-sen University, Guangzhou 510275, China.

^3^ Dongguan Institute of Materials Science and Technology, Chinese Academy of Sciences, Dongguan 523808, China.

^4^ Neutron Science Center, Songshan Lake Materials Laboratory, Dongguan 523808, China.

^5^ Sino-French Institute of Nuclear Engineering and Technology, Sun Yat-Sen University, Zhuhai 519000, China.

^6^ Shanghai Synchrotron Radiation Facility, Shanghai Advanced Research Institute, Chinese Academy of Sciences, Shanghai 201204, China.

^7^ Department of Physics, City University of Hong Kong, 83 Tat Chee Ave., Hong Kong, China.

^8^ Southern Marine Science and Engineering Guangdong Laboratory, Zhuhai 519000, China.

^9^ School of Materials Science and Engineering, Sun Yat-sen University, Guangzhou 510275, China.

(Wei Chen and Xuerui Wei contributed equally to this work.)

* Corresponding author: luzhichao@dimst.ac.cn (Z.C. Lu), mengfq5@mail.sysu.edu.cn (F.Q. Meng), zhouzh68@mail.sysu.edu.cn (Z. Zhou), dongma@sslab.org.cn (D. Ma).

**Figure S1** Experimental setup and simulation method. (a) Schematic illustration of in situ high-energy synchrotron X-ray diffraction study of aerodynamically levitated Ti-Cu melts. The red solid ball represents the nearly 2-mm-diameter spherical sample levitated during the experiment, with interchangeable nozzles of different apertures mounted beneath. The right panel shows a typical two-dimensional detector image collected in 0.2 s. (b) Flowchart of the energy minimization-enhanced reverse Monte Carlo (EM-RMC) simulation method.

**Figure S2** DSC curves measured at various heating rates for (a) Ti_27_Cu_73_ and (b) Ti_50_Cu_50_ glasses.

**Table S1.** Crystallization temperatures ($T_{x}$) of Ti-Cu MGs measured at different heating rates

| MG | 5 K/min | 10 K/min | 20 K/min | 30 K/min | 40 K/min |
| --- | --- | --- | --- | --- | --- |
| Ti_27_Cu_73_ | 611.9 K | 621.9 K | 632.7 K | 639.2 K | 643.1 K |
| Ti_50_Cu_50_ | 667.7 K | 671.9 K | 679.8 K | 683.7 K | 687.7 K |

**Figure S3** Synchrotron X‑ray structure factors $S(q)$ of aerodynamically levitated Ti–Cu melts at different reduced temperatures: (a) Ti_27_Cu_73_, (c) Ti_50_Cu_50_. Dashed lines are guides to the eye, highlighting the evolution of the second peak from well‑separated to smeared out with increasing temperature. Using the $S\left( q \right)$ curve at $T/T_{l}=1.02$ as a baseline, we calculated the differences for the $S\left( q \right)$ profiles at various temperatures within the range of the second peak and its shoulder, thereby magnifying their temperature-dependent evolutionary trends: (b) Ti_27_Cu_73_, (d) Ti_50_Cu_50._

**Figure S4** EM-RMC reconstructed three-dimensional configurations of the (a) Ti_27_Cu_73_ and (b) Ti_50_Cu_50_ melts at $T/T_{l}=1.02$.

**Figure S5** Relationship between the cluster variance ($\sigma^{2}$) and Shannon entropy ($S_{\mathrm{sh}}$) in Ti–Cu alloy melts.

**Figure S6** Experimentally determined reduced pair distribution functions $G(r)$ of Ti_27_Cu_73_ and Ti_50_Cu_50_ glasses, respectively, compared with those obtained from EM-RMC reconstructed configurations.

**Figure S7** Atomic maps showing the spatial distribution of central atoms of $\left\langle0,0,12,0 \right\rangle$, $\left\langle0,1,10,2 \right\rangle$ and $\left\langle0,1,10,3 \right\rangle$clusters in the EM-RMC reconstructed glass configurations, highlighting the connectivity of high fivefold-symmetric clusters by node degree K: (a) Ti_27_Cu_73,_ (b) Ti_50_Cu_50_.

**Figure S8** (a) Equilibrium phase diagram of the Ni–Nb binary alloys [1]. (b) Dependence of the GFA parameter $\gamma^{*}$ on the compositions of Ni–Nb alloys [2].

**Note S1**

We used MD to generate high-temperature melt structures of Ni_58.5_Nb_41.5_ and Ni_75_Nb_25_ at different temperatures ($T/T_{l}$=1.02, 1.04, 1.06 and 1.08). The details are consistent with those used to construct the initial Ti–Cu configurations in the Methods section of the manuscript. As shown by the Ni–Nb binary phase diagram and the γ* criterion in Figure S8, the GFA of the eutectic composition Ni_58.5_Nb_41.5_ is significantly better than that of the congruently melting Ni_75_Nb_25_. Using the same criteria as in the main text, we compared the melt structural complexity of the two compositions.

First, we calculated the evolution of the cluster fraction variance ($\sigma^{2}$) and Shannon entropy ($S_{\mathrm{sh}}$) of the Ni–Nb alloy melts with temperature. As shown in Figure S9a, $\sigma^{2}$for Ni_58.5_Nb_41.5_ is always lower than that for Ni_75_Nb_25_, while its $S_{\mathrm{sh}}$remains consistently higher than that of Ni_75_Nb_25_. This indicates that the high-temperature melt of Ni_58.5_Nb_41.5_ has a more uniform distribution of cluster types and populations, reflecting higher cluster diversity.

Second, we further introduced the cluster correlation matrix ($C_{ij}$) to characterize the spatial correlations among different types of clusters in the melt. As shown in Figures S9b and S9c, at $T/T_{l}$=1.02, clusters with high fivefold symmetry ($f^{5}$) in the Ni_58.5_Nb_41.5_ high-temperature melt exhibit more pronounced correlation features than those in Ni_75_Nb_25_. In addition, using the variance of cluster correlations ($\sigma_{C_{ij}}^{2}$) proposed in the main text, we systematically evaluated the overall degree of correlation for the two compositions at each temperature. As shown in Figure S9d, $\sigma_{C_{ij}}^{2}$for Ni_58.5_Nb_41.5_ is always higher than that for Ni_75_Nb_25_, further confirming stronger spatial connectivity and coupling among its clusters.

Taken together, Ni_58.5_Nb_41.5_ not only possesses richer cluster diversity but also exhibits stronger cluster correlations, which corroborates the complexity of its melt structure at the atomic scale.

**Figure S9** Cluster diversity and cluster correlation in high-temperature melts of Ni-Nb alloys. (a) Temperature dependence of the cluster fraction variance ($\sigma^{2}$) and Shannon entropy ($S_{sh}$). (b) Cluster correlation index of the Ni_58.5_Nb_41.5_ high-temperature melt ($T/T_{l}$=1.02). (c) Cluster correlation index of the Ni_75_Nb_25_ high-temperature melt ($T/T_{l}$=1.02). (d) Temperature dependence of the cluster correlation variance.

**Figure S10** Photographs of the aerodynamic levitation setup at BL12SW of the Shanghai Synchrotron Radiation Facility: (a) front view; (b) rear view, together with an enlarged view of the nozzle. The orange Kapton film seals the furnace chamber and effectively reduces background scattering.

**Figure S11** 2D contour maps and selected 1D diffraction patterns during the melting process of Ti-Cu alloys: (a, b) Ti_27_Cu_73_, and (c, d) Ti_50_Cu_50_.

**Figure S12** Comparison of the results from conventional RMC and EM-RMC against the target configuration. Reduced pair distribution functions $G(r)$: (a) Conventional RMC and (b) EM-RMC. Pair distribution functions $g(r)$: (c) Conventional RMC and (d) EM-RMC.

**Figure S13** Comparison of the results from conventional RMC and EM-RMC with the target configuration. (a, b) Partial pair distribution functions $g_{ij}(r)$ for RMC and EM-RMC, respectively; (c, d) Corresponding deviations.

**Figure S14** Typical $\delta$-landscape as a function of $N$ and $M$ obtained from EM-RMC simulations for (a, c) Ti_27_Cu_73_ and (b, d) Ti_50_Cu_50_, respectively. The red dashed circles mark the minimum $\delta$ value, corresponding to the optimal atomic configuration resolved by EM-RMC. Specifically, the total number of simulation steps was set to 9,000,000 for the high-temperature melts and 20,000,000 for the glasses.

**References**

1. H. Okamoto, “Nb-Ni (Niobium–Nickel),” *Journal of Phase Equilibria and Diffusion* *29*, no. 2 (2008): 210–211, https://doi.org/10.1007/s11669-008-9277-0.

2. L. Xia, W. H. Li, S. S. Fang, B. C. Wei, and Y. D. Dong, “Binary Ni–Nb Bulk Metallic Glasses,” *Journal of Applied Physics* *99*, (2006): 026103, https://doi.org/10.1063/1.2158130.
